# The Protective Effect of Exogenous 17β-Estradiol against Experimentally Induced Oxidative Damage to Membrane Lipids Is Stronger in Male vs. Female Porcine Thyroids: Preliminary Results

**DOI:** 10.3390/toxics11090746

**Published:** 2023-09-01

**Authors:** Jan Stępniak, Edward Koziróg, Małgorzata Karbownik-Lewińska

**Affiliations:** 1 Department of Oncological Endocrinology, Medical University of Lodz, 7/9 Zeligowski St., 90-752 Lodz, Poland; jan.stepniak@umed.lodz.pl (J.S.); edward.kozirog@gmail.com (E.K.); 2 Polish Mother’s Memorial Hospital–Research Institute, 281/289 Rzgowska St., 93-338 Lodz, Poland

**Keywords:** 17β-estradiol, thyroid, oxidative stress, Fenton reaction, sexual dimorphism

## Abstract

It is well-known that thyroid diseases are more prevalent in women than in men. The contribution of sex hormones may explain such disparity. The aim of this study was to check if there are any differences between sexes concerning the effects of 17β-estradiol on oxidative damage to membrane lipids (lipid peroxidation) in porcine thyroid homogenates under basal conditions and in the presence of Fenton reaction (Fe^2+^ + H_2_O_2_→Fe^3+^ + ^•^OH + OH^−^) substrates. We observed that 17β-estradiol did not change the basal level of lipid peroxidation (measured spectrophotometrically as concentrations of malondialdehyde + 4-hydroxyalkenals) in thyroid homogenates, and no differences were found between sexes. The lipid peroxidation level in response to Fe^2+^ + H_2_O_2_ plus 17β-estradiol was lower in male thyroids. In turn, in male thyroids, 17β-estradiol reduced experimentally induced lipid peroxidation in as low of a concentration as 0.1 μM, whereas in female thyroids the lowest effective concentration of 17β-estradiol was 10 μM, i.e., 100 times higher than in males. In conclusion, the protective effects of exogenous 17β-estradiol against experimentally induced oxidative damage to membrane lipids is stronger in male than in female thyroids. Our observation suggests that female tissue is less sensitive to the protective effects of exogenous 17β-estradiol. This sexual dimorphism of oxidative processes in the thyroid may constitute one of the mechanisms of the different prevalence of thyroid diseases in women and in men.

## 1. Introduction

The presence of sex differences in the incidence, prevalence and severity of many human diseases is well documented. This phenomenon affects a wide spectrum of disorders such as cancer, diabetes and neurological, cardiovascular and autoimmune diseases [1,2,3]. Regarding malignancies, women generally have a lower risk than men [4]. A notable exception is thyroid cancer, which is female-dominant and is 3–4 times more likely to develop in women than in men [4,5,6]. This gender disparity also applies to the incidence of autoimmune thyroid diseases, such as Hashimoto’s thyroiditis, as well as—in iodine deficient areas—of diffuse or nodular goiter [7]. Moreover, the detection of thyroid autoantibodies is almost five times more common in women than in men [8]. These observations clearly indicate that thyroid diseases are more prevalent in women than in men.

Despite the importance of sex dimorphism in case of thyroid diseases, the biological factors and mechanisms that drive these differences are still poorly understood. However, evidence from an increasing number of studies shows that sex hormones—mainly estrogens—may be responsible for this phenomenon. Such a hypothesis is supported, among others, by the recent observation that the genetic sex-specific molecular profile did not differ between women and men with well-differentiated thyroid cancer [9].

In the context of the parameters of oxidative damage measured in the present study, it should be mentioned that gender differences in oxidative stress in relation to diseases are documented [10].

17β-Estradiol—the most biologically prevalent and active estrogen—has been shown to have a strong proliferative effect on the primary culture of thyroid cells, and it could have a role in the pathogenesis of thyroid cancer [11]. Moreover, it has been shown that 17β-estradiol may play a crucial pathogenic role in thyroid diseases via oxidative mechanisms, as it can stimulate H_2_O_2_ production [12]. Another potential mechanism that may have a negative effect on the thyroid gland is potentially associated with the direct action of estrogen metabolites. This mechanism is independent of the interaction between a hormone and its receptor and relies on oxidative damage to macromolecules by the semiquinone and quinone metabolites [13].

On the other hand, 17β-estradiol may also reveal anti-oxidant action which can be mediated in a number of ways, such as the enhancement of the cellular anti-oxidative defense molecules like SOD, GPx1 and GPx4, or by the direct neutralization of the excess of reactive oxygen species (ROS). This direct action of 17β-estradiol occurs without any involvement of estrogen receptors and results from the presence of the phenolic hydroxyl group at the C3 position on the A ring of the steroid molecule. Such a chemical structure is similar to that of simple phenolic anti-oxidants, such as vitamin E or butylated hydroxytoluene [14].

In fact, in our earlier studies, we have shown that 17β-estradiol at concentrations close to physiological level prevents experimentally induced oxidative damage to membrane lipids (lipid peroxidation; LPO) and oxidative damage to nuclear DNA in porcine ovary, most likely as a result of its direct action [15]. In our other study, it has been shown that this hormone may contribute to minimizing the negative effects of iron overload, as exogenous 17β-estradiol reduced experimentally induced LPO independently of iron concentration in porcine thyroid and ovary homogenates [16].

All of the above findings suggest the diverse effects of 17β-estradiol, i.e., anti-oxidative and pro-oxidative action. Taking into consideration the aforementioned potential contribution of this hormone to the events that may result in the gender disparity of thyroid disorders, a need arises for studies concerning gender-specific oxidative stress in response to 17β-estradiol.

Hence, the aim of this study was to assess the influence of 17β-estradiol on the oxidative damage to membrane lipids in porcine thyroid homogenates under basal conditions and in the presence of Fenton reaction substrates (a frequently used model of experimentally induced oxidative damage [15,16,17]) and to check if there are any differences between sexes concerning these effects.

## 2. Materials and Methods

### 2.1. Chemicals

17β-Estradiol, ferrous sulfate (FeSO_4_) and hydrogen peroxide (H_2_O_2_) were purchased from Sigma (St. Louis, MO, USA). The LPO-586 kit for lipid peroxidation was obtained from Enzo Life Science (Farmingdale, NY, USA). All the used chemicals were of analytical grade and came from commercial sources.

### 2.2. Animals

Porcine thyroids were collected from 21 male and 21 female animals at a slaughter-house. Animals were treated according to the European Community Council Regulation (CE1099/2009) concerning the protection of animals at the time of killing. All animals were sexually mature as determined by age (8–9 months) and body mass [118.13 ± 4.2 (SD) kg]. They were in good body condition and considered free of pathologies by the veterinary medical officer responsible for the health of the animals and the hygiene of the slaughterhouse.

The mean mass of the collected thyroid was 32 ± 1.5 (SD) g. Collected thyroids were frozen on solid CO_2_ and stored at −80 °C until the assay.

### 2.3. Assay of Lipid Peroxidation

Thyroid tissue was homogenized in ice-cold 20 mM Tris-HCl buffer (pH 7.4) (10%, *w*/*v*) and then incubated for 30 min at 37 °C in the presence of examined substances.

In the first set of experiments, homogenates of male or female thyroids were incubated in the presence of 17β-estradiol only (1 mM; 100 μM; 10 μM; 1 μM; 100 nM; 10 nM; 1 nM; 100 pM; 10 pM; 1 pM).

In the second set of experiments, homogenates of male or female thyroids were incubated in the presence of 17β-estradiol (1 mM; 100 μM; 10 μM; 1 μM; 100 nM; 10 nM; 1 nM; 100 pM; 10 pM; 1 pM) with the addition of Fenton reaction substrates, i.e., FeSO_4_ (30 µM) plus H_2_O_2_ (0.5 mM). The reactions were stopped via cooling the samples on ice. Each separate experiment was repeated independently three times. Therefore, for each sex, three tissue pools were prepared, with seven (7) thyroid glands used for each homogenate pool.

### 2.4. Measurement of Lipid Peroxidation Products

The concentration of malondialdehyde + 4-hydroxyalkenals (MDA + 4-HDA), as an index of LPO, was measured in tissue homogenates, as described elsewhere [18]. Protein was measured using the method of Bradford [19]. The level of lipid peroxidation was expressed as the amount of MDA + 4-HDA (nmol) per mg protein and additionally as the amount of MDA + 4-HDA (nmol) per mg tissue.

### 2.5. Statistical Analyses

The data were statistically analyzed, using a one-way analysis of variance (ANOVA), followed by the Student–Neuman–Keuls’ test, or using an unpaired *t*-test. Statistical significance was determined at the level of *p* < 0.05. Results are presented as means ± SE.

## 3. Results

There was no difference in protein concentration between male and female thyroid homogenates under basal conditions (6.505 vs. 6.781 mg/mL, *p* = 0.444).

**Figure 1:**
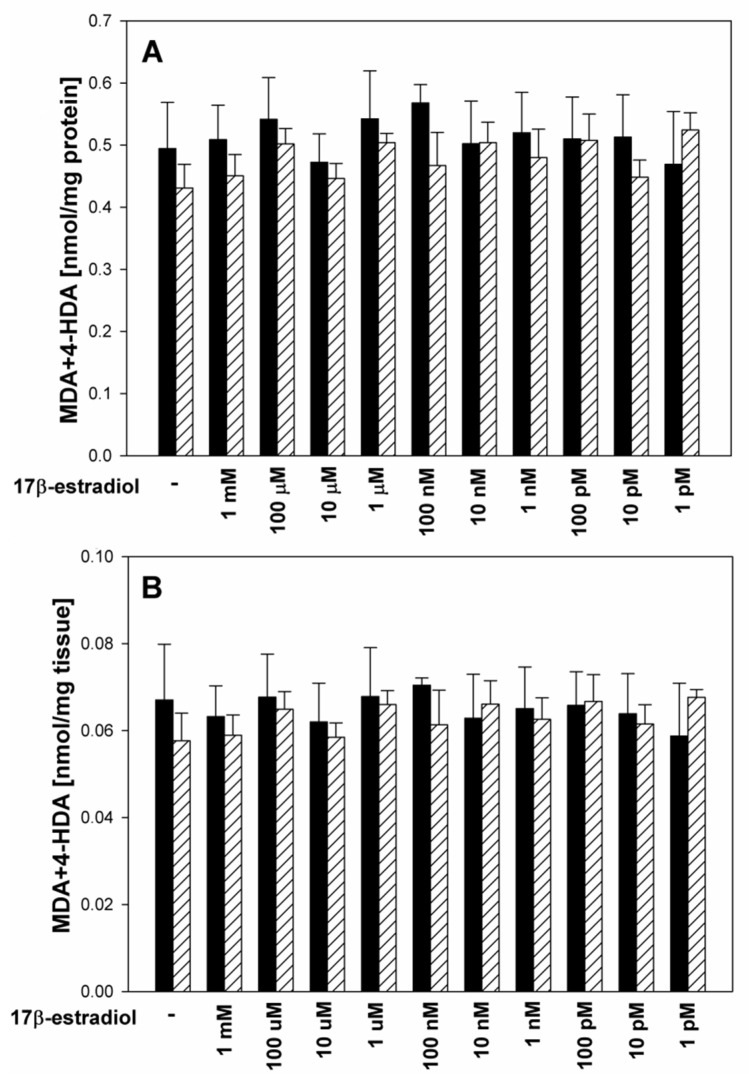
Lipid peroxidation, measured as MDA + 4-HDA level, and expressed in nmol per mg protein (**A**) or in nmol per mg tissue (**B**) in male (black bars) and female (striped bars) porcine thyroid homogenates. Homogenates were incubated in the presence of 17β-estradiol alone (1 mM; 100 μM; 10 μM; 1 μM; 100 nM; 10 nM; 1 nM; 100 pM; 10 pM; 1 pM). Bars represent the mean ± SE of three independent experiments run in duplicates. No significant differences were found.

In the first set of experiments, 17β-estradiol did not change the basal level of lipid peroxidation in thyroid homogenates, and no differences were observed between sexes (Figure 1A,B).

**Figure 2:**
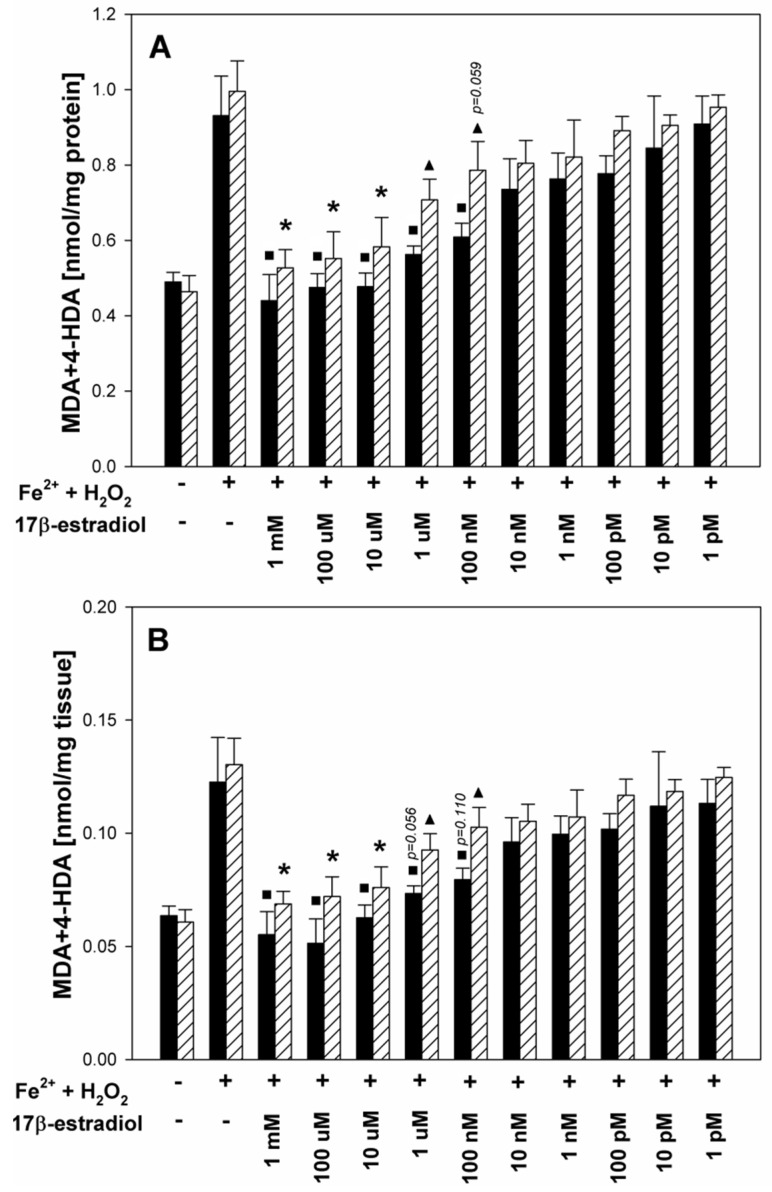
Lipid peroxidation, measured as MDA + 4-HDA level, and expressed in nmol per mg protein (**A**) or in nmol per mg tissue (**B**) in male (black bars) and female (striped bars) porcine thyroid homogenates. Homogenates were incubated in the presence of 17β-estradiol (1 mM; 100 μM; 10 μM; 1 μM; 100 nM; 10 nM; 1 nM; 100 pM; 10 pM; 1 pM) together with Fenton reaction substrates [FeSO_4_ (30 μM) + H_2_O_2_ (0.5 mM)]. Bars represent the mean ± SE of three independent experiments run in duplicates. ^■^ *p* < 0.05 vs. Fe^2+^ + H_2_O_2_ (in the absence of 17β-estradiol) in male thyroid; * *p* < 0.05 vs. Fe^2+^ + H_2_O_2_ (in the absence of 17β-estradiol) in female thyroid; ^▲^ *p* < 0.05 vs. the same concentration of 17β-estradiol in male thyroid.

In the second set of experiments, male and female porcine thyroid tissue revealed similar responses to Fenton reaction substrates, i.e., Fe^2+^ (30 µM) plus H_2_O_2_ (0.5 mM) increased lipid peroxidation to a similar extent in both sexes. In turn, 17β-estradiol added to either male or female porcine thyroid homogenates together with Fe^2+^ (30 µM) plus H_2_O_2_ (0.5 mM) decreased—in a concentration-dependent manner—lipid peroxidation induced by the Fenton reaction (Figure 2).

However, in male thyroid homogenates, protective effects of 17β-estradiol was observed for its five highest concentrations, i.e., 1 mM, 100 μM, 10 μM, 1 μM, 100 nM, while in female thyroids, 17β-estradiol reduced experimentally induced oxidative damage to membrane lipids only at the three highest concentrations, i.e., 1 mM, 100 μM, 10 μM. Thus, the lowest effective concentration of 17β-estradiol in male thyroid homogenates was 100 times lower than that in female thyroid homogenates (Figure 2A). These differences in the protective effects of 17β-estradiol were also noticeable when LPO was expressed as the amount of MDA + 4-HDA (nmol) per mg tissue, however, with borderline statistical significance (Figure 2B).

Moreover, LPO levels in response to Fe^2+^+H_2_O_2_ plus 17β-estradiol were lower in male thyroids (Figure 2A,B); however, these differences reached statistical significance only for the two 17β-estradiol concentrations of 1 μM and 100 nM (Figure 2A,B).

## 4. Discussion

Estrogens—with the most biologically prevalent and active 17β-estradiol—are natural steroid hormones with various physiological actions. Previous research shows that they can act in various tissues as pro-oxidants as well as anti-oxidants. In the present study, we have found that 17β-estradiol prevents Fenton reaction-induced oxidative damage to membrane lipids in porcine thyroid. These results are in line with our earlier observations; namely, in porcine ovaries, this estrogen also prevented oxidative damage to membrane lipids induced by the same concentrations of Fenton reaction substrates [FeSO_4_ (30 μM) + H_2_O_2_ (0.5 mM)] [15]. However, in the ovary, 17β-estradiol showed protective anti-oxidative properties at lower concentrations than in the thyroid (10 vs. 100 nM), which may indicate a greater susceptibility of the ovary to the protective effects of this hormone. In another study, we have found that 17β-estradiol protects against Fenton reaction-induced lipid peroxidation in ovary and thyroid homogenates, when iron was used in concentrations that were two orders of magnitude higher [16]. The protective effect of 17β-estradiol was independent of iron concentration and was observed for the three highest-used concentrations of 17β-estradiol, i.e., 1 mM, 100 µM, and 10 µM, which consistently reveal protective anti-oxidative action in our previous studies [15,16] and also in the present study.

All these findings, which indicate the anti-oxidative role of 17β-estradiol, support the point of view that this hormone plays an important role in the response to enhanced oxidative stress. This role can be particularly evident in cases of gender-related disorders. It has been shown that in females, menopause creates a systemic pro-oxidant state due to decreased production of estrogens. This enhanced oxidative stress is directly related to an increased risk of cardiovascular diseases [20] and to a decline in endothelial function [21]. An experimental study in dogs has shown that a decline in 17β-estradiol alters anti-oxidant enzyme activity in serum and that these changes are associated with an increase in body mass [22]. A study in mice has shown that estrogens protect female mice from developing oxidative stress within adipose tissue [23]. Moreover, in the present study, we have observed that 17β-estradiol does not affect the basal level of lipid peroxidation in the thyroid gland in both male and female homogenates (Figure 1). This effect is in fact favorable as the thyroid gland requires a certain level of oxidative stress for normal functioning, and a reduction in ROS below this level could lead to disturbances in the functioning of this gland [24].

Considering the results of our study and those cited above, one can conclude that 17β-estradiol can have a fully beneficial effect on the oxidative status of the thyroid gland. On the other hand, however, in our previous study, carried out on primary cell cultures derived from the thyroid glands of adult male or female Wistar rats, we have shown that exogenous 17β-estradiol has a major negative impact on the redox state of thyroid cells; this effect is induced through the stimulation of NADPH oxidase expression and, consequently, stimulation of H_2_O_2_ production [12]. A similar result was obtained in PCCL3 rat thyroid follicle cells [25]. However, it should be stressed that the concentrations of 17β-estradiol used in the above cited studies [12,25] were relatively low (from 1 to 100 nM).

These seemingly contradictory results showing pro-oxidative or anti-oxidative effects of 17β-estradiol are in agreement with the general hypothesis that in contrast to high levels of 17β-estradiol, low levels of 17β-estradiol might have a pro-oxidant-like effect. It has been postulated that the pro/anti-oxidative effect of 17β-estradiol is largely dependent on the specificity of the cell in which estrogens generate their hormone response [26]. It should be stressed that there is still a debate in the literature regarding the consequences of the pro-oxidative effects of estrogens, such as possible carcinogenic action [27].

The main observation from the present study is that in male thyroids, protective effects against experimentally induced lipid peroxidation were caused by lower concentrations of 17β-estradiol when compared to female thyroids. Additionally, the lipid peroxidation level in response to Fe^2+^ + H_2_O_2_ plus 17β-estradiol was again lower in male than in female thyroids. Such an observation suggests the higher sensitivity of male tissue to the protective anti-oxidative effects of exogenous 17β-estradiol. This phenomenon can be explained by the fact that throughout its life, the female thyroid is exposed to much higher 17β-estradiol concentrations than the male thyroid, resulting in a kind of adaptation of female tissues to hyperestrogenism. Therefore, the male thyroid may be more sensitive to increased 17β-estradiol levels, associated with any additional conditions, especially resulting from the exposure to exogenous hormones.

It should be stressed that concentrations of 17β-estradiol, which revealed protective effects in the present study, can only be achieved under experimental conditions. The highest physiologically achievable concentration of 17β-estradiol is estimated to be approx. 25 nM [28]. However, it should be underlined that in vitro conditions differ substantially from those in vivo in such a sense that the latter is always associated with the exposure to complex network of numerous endogenous factors. Although in vitro effects may not be directly extrapolated into in vivo conditions, a certain direction of action of any factor observed in vitro should also be considered in living organisms. So, the evidence in favor of anti-oxidative protection by 17β-estradiol obtained in the present study may explain, at least partially, the protection observed with the physiological level of estrogens in vivo.

Taking into account the experimental model we have used in this study, i.e., tissue homogenates, we can also conclude that the anti-oxidative effect of 17β-estradiol resulted most likely from a direct neutralization of excess ROS. Moreover, in the case of homogenates, we should rather not expect strong adverse effects from high 17β-estradiol concentrations. It is due to the fact that in such an environment, the metabolism of this hormone is limited, and thus the formation of its more harmful pro-oxidative metabolites, such as semiquinone and quinone, is restricted.

It should be stressed that the present study is the first one showing sex differences regarding the protective effects of 17β-estradiol against experimentally induced lipid peroxidation. However, because it is a preliminary one, further research is required with the use of other experimental models, possibly other species, and with the examination of other biological molecules, such as DNA and proteins. As natural anti-oxidants have recently been recommended to eliminate the toxic effects of radioiodine used in the treatment of thyroid cancer [29], 17β-estradiol should be considered as a prominent candidate applied in studies on protection against the damaging effects caused by radioiodine or by other kinds of treatment. However, taking into account the potential pro-oxidative, and, therefore, unfavorable, action of 17β-estradiol, its simultaneous application with other anti-oxidants should be considered in future studies, especially since a cumulative protective effect of two anti-oxidant indole substance has recently been observed in the thyroid gland [30].

## 5. Conclusions

In conclusion, whereas exogenous 17β-estradiol exhibits protective effects against experimentally induced oxidative damage to membrane lipids, in the present study, we have observed for the first time that this effect is stronger in male than in female thyroids. Our observation suggests that female tissue is less sensitive to the protective effects of exogenous 17β-estradiol. This sexual dimorphism of oxidative processes in the thyroid may constitute one of the mechanisms of the higher prevalence of thyroid diseases in women than in men.

## Data Availability

The datasets used and/or analyzed during the current study are available from the corresponding author on reasonable request.
